# Relationship between clinicopathologic factors and FDG avidity in radioiodine-negative recurrent or metastatic differentiated thyroid carcinoma

**DOI:** 10.1186/s40644-020-00378-z

**Published:** 2021-01-07

**Authors:** Le Ngoc Ha, Amir Iravani, Nguyen Thi Nhung, Ngo Thi Minh Hanh, Febby Hutomo, Mai Hong Son

**Affiliations:** 1 Department of Nuclear Medicine, 108 Central Military Hospital, 1st Tran Hung Dao, Hai Ba Trung, Ha Noi, Vietnam; 2 Washington University School of Medicine, Mallinckrodt Institute of Radiology, St Louis, MO USA; 3 Department of Pathology, 108 Central Military Hospital, Hanoi, Vietnam; 4 Nuclear Medicine Department, MRCCC Siloam Hospital, Jakarta, Indonesia

**Keywords:** ^18^F-fluorodeoxyglucose, BRAF ^V600E^ mutation, Histopathologic type, Radioiodine-refractory, Differentiated thyroid carcinoma

## Abstract

**Background:**

In this study, we investigated the relationship between clinicopathologic factors, BRAF^V600E^ mutation status and [^18^F] F-fluoro-2-deoxyglucose (FDG) avidity in patients with radioiodine (RAI)-negative recurrent or metastatic differentiated thyroid cancer (DTC).

**Methods:**

From 2015 to 2018 all patients with suspected recurrent or metastatic radioiodine-negative DTC patients who underwent FDG positron emission tomography/computed tomography (PET/CT) were retrospectively reviewed. Suspected lesions on FDG PET/CT were biopsied and underwent BRAF^V600E^ mutation testing by immunohistochemistry and real-time PCR. Tumor size, recurrent versus metastatic disease, histopathologic features including classical type versus aggressive subtypes (poorly differentiated, tall cell, columnar cell, hobnail variants) and BRAF^V600E^ mutation status were correlated with the SUVmax of highest hypermetabolic lesions on FDG PET/CT by the univariate analysis using logistic regression.

**Results:**

Sixty-three consecutive patients, 55 (87.3%) female, with median age of 48 (range 17–81) were included. The majority of patients had BRAF^V600E^ mutation and classical subtype, 55/63 (87.3%) and 45/63(71.4%), respectively. Thyroglobulin at the time of suspected recurrence was 262.7 ng/ml (range 16.3–1000) and patients received a median 3 prior RAI treatments. Fifty-four patients (85.7%) had local recurrence. The majority of patients 58/63 (92.1%) had FDG-avid disease on PET/CT. On univariate analysis, tumor size aggressive histopathologic types and distant metastasis are the significant factors for predicting FDG uptake, *p* = 0.04, *p* = 0.001 and *p* = 0.004 respectively. Although FDG uptake of BRAF^V600E^ bearing recurrent/metastatic RAIR DTC lesions was higher than those without the mutation, the difference did not reach statistical significance, SUVmax of 7.11 versus 4.91, respectively, *p* = 0.2.

**Conclusion:**

The majority of recurrent or metastatic RAI-negative DTC have BRAF^V600E^ mutation and detectable disease on FDG PET/CT. FDG avidity of the recurrent or metastatic RAI-negative DTC is independently associated with the aggressive histopathologic features.

## Key points

Question: Is there a relationship between clinicopathological features of recurrent or metastatic RAI-negative DTC and FDG-avidity on PET/CT?

## Introduction

Thyroid carcinoma is one of the most popular endocrine cancer worldwide. Differentiated thyroid cancer (DTC) accounts for 90% of all thyroid cancer types [1]. Approximately 5% of patients with DTC follow a more aggressive course with radioiodine (RAI)-refractory or RAI-negative disease, often becoming the cause of mortality associated with tumor recurrences and distant metastases [2]. These DTC patients were predicted to have poorer prognosis and limited effective treatments including surgery, radiation therapy, chemotherapy, immunotherapy and tyrosine kinase inhibitors [3]. As a result, risk stratification and prognostic evaluation are required to identify high-risk patients and guide the appropriate treatment modality.

Histopathologic and biomolecular markers play an important role in the improvement of risk stratification in DTC. Histopathologic subtype of thyroid cancer is grouped as classical subtype (well-differentiated thyroid carcinoma) and aggressive subtype (poorly differentiated, tall cell, columnar cell, hobnail variants of DTC) [4] . Classical subtype, characterized by a papillary and follicular variant, has an excellent prognosis, while patients with aggressive histopatholologic features in the primary tumor are considered at risk of developing RAI-refractory DTC [5]. BRAF^V600E^ is the most common mutation observed in DTC and triggers tumorigenesis through the mitogen-activated protein kinase (MAPK) pathway [6]. Multiple studies have shown BRAF^V600E^ mutation is associated with poor clinicopathologic outcomes, larger tumor size, local recurrence and distant metastases [7, 8].

[^18^F] F-fluorodeoxyglucose (FDG) positron emission tomography/computed tomography (PET/CT) is a noninvasive diagnostic modality and beneficial for localizing residual or recurrent disease, particularly when iodine avidity of disease has been lost (RAI-negative) [9, 10]. The level of metabolic activity of the disease on FDG PET/CT is independently associated with the patient survival, hence may guide to individualize the intensity of follow-up and treatment of these patients [11, 12]. A limited number of studies have investigated the association between FDG-avidity of the tumors with BRAF^V600E^ mutation status and clinicopathological features [13–15]. While some studies suggest primary or recurrent tumors bearing BRAF^V600E^ mutation may have higher FDG-avidity [16, 17], data on the correlation between other clinicopathological features and FDG-avidity are rather sparse. Therefore, we aimed to determine the relationship between clinicopathologic factors, BRAF^V600E^ mutation status and FDG avidity in a rather homogenous group of patients with recurrent or metastatic RAI-negative DTC.

## Methods

### Patients

This is a retrospective review of all patients from 2015 to 2018 who had 1) prior RAI ([^131^I] treatment, 2) had negative diagnostic or post-treatment iodine scan following suspected recurrent disease based on rising thyroglobulin (Tg) or ultrasound findings, 3) underwent FDG PET/CT for detection of the site of recurrence. The suspected site of recurrence on either FDG PET or contrast-enhanced component of PET/CT were biopsied with BRAF^V600E^ testing on the sample.

The present study protocol was reviewed and approved by the Institutional Review Board of Viet Nam Ministry of Science and Technology (approval No. 02/HĐTK-ĐTCT-KC.10.03/16–20).

### Procedures

PET/CT examination was performed, using GE Discovery 710, according to the European Association of Nuclear Medicine (EANM) guidelines, version 1.0 [18]. For patient preparation, the serum glucose level was checked to exclude hyperglycemia. Afterward, the patients rested in the waiting room before intravenous injection of 2.5 MBq/kg body weight (±10%) of FDG. Contrast-enhanced CT with 100 ml of the contrast material with a scan delay of 30 s and an injection rate of 3 ml/s from the skull base to the mid-thigh was performed 60 min after FDG injection. The parameters of the CT scan were as follows: 120 kVp, 100 mA, the helical thickness of 3.75 mm and 0.5 s/rotation. PET images were reconstructed using an iterative algorithm with attenuation correction with CT.

Histopathologic type of thyroid cancer was divided into classical type (well-differentiated thyroid carcinoma) and aggressive histologic types (poorly differentiated, tall cell, columnar cell, hobnail variants of DTC). BRAF^V600E^ mutation was analyzed by using immunohistochemistry and real-time PCR. The immunohistochemical method was performed using anti-BRAF^V600E^ (VE1) antibody (Ventana Medical System) on automated BenchMarch Ultra (Ventana Medical System, USA). DNA for real-time PCR was extracted from formalin-fixed, paraffin-embedded tissue obtained from core needle biopsy (*n* = 44) and percutaneous needle aspiration (*n* = 19) specimens. Each DNA sequence was read on an ABI-PRISM 3100 automatic sequencer (Applied Biosystems) in order to determine the presence of the BRAF mutation. The positive BRAF^V600E^ mutation was decided by the concordance between the histopathologist and molecular biologist in 108 hospital.

### Qualitative and semiquantitative assessment

FDG PET/CT images were evaluated by two nuclear medicine physicians (NMPs) blinded to the clinical data. FDG-avid lesions are defined as uptake above that of mediastinal blood pool activity by the consensus of two NPMs. Semiquantitative analysis of highest hypermetabolic lesion, determined by maximum standardized uptake value (SUVmax) was assessed by automated polygonal regions of interest (ROIs) drawing on attenuation-corrected PET images using the GE workstation (version 4.7, GE Healthcare). For suspected malignant lesions on contrast-enhanced CT with no visual FDG uptake on PET, the manual ROIs were drawn on CT and cloned to co-registered PET images to record the SUVmax. In cases of multiple malignant lesions, an ROI was drawn on a lesion with the highest SUVmax on PET.

### Statistical analysis

Categorical values were compared utilizing the Chi-squared test or Fisher’s exact test. Continuous variables following normal distribution were compared with paired t-test or repeated measure ANOVA and for variables not following a normal distribution with Wilcoxon signed-rank test or Friedman test. To analyze the relationship between clinicopathologic variables and SUVmax, the univariate analysis was performed by logistic regression. The significance threshold was set at *P* ≤ 0.05. The statistical software Statistical Package for the Social Sciences (SPSS) 20.0 (SPSS Inc., Chicago IL, USA) and GraphPad Prism (version 8.0 Graphpad Software, Inc., USA) have been used to analyze the data.

## Results

### Patients characteristics

Sixty-three consecutive patients, 55(84.8%) female, and 8 (15.2%) male, with the median age of 48 (range,17–81), were included in the study. The patients had received median 3 doses (range 1–9) of I-131 and the median thyroglobulin (Tg) at the time of suspected recurrence was 262.7 ng/ml (Table 1). The majority of the patients, 55/63 (89%), had BRAF^V600E^ mutation. In regard to histopathologic variants, the proportion of classical DTC was 45/63 (71.4%) and higher than those of aggressive subtypes 18/63 (28.6%). There was no significant difference in the prevalence of BRAF^V600E^ mutation by the histopathologic subtype of DTC, 40/45 (88.9%) in classical subtype vs. 15/18 (83.3%) in aggressive subtype (*P* = 0.6), respectively (Table 2).

**Table 1:** Clinicopathologic features of radioiodine-negative DTC patients

Clinicopathologic features	Number (*n* = 63)	Percent(%)
Age (median, range)	48 (17–81)	
*≤ 45*	31	49.2
*> 45*	32	50.8
Gender		
*Male*	8	12.7
*Female*	55	87.3
Cumulative I-131administered activity		
*< 600 mCi*	59	93.7
*≥ 600 mCi*	4	6.3
Number of I-131 treatment, median (range)	3 (1–9)	
Serum Tg level (ng/ml), median (range)	262.7 (16.3–1000)	
Location of lesion recurrence/metastases		
*Thyroid bed alone*	2	3.2
*Cervical lymph node alone*	44	69.8
*Distant metastasis alone*	1	1.6
*Thyroid bed and regional lymph node*	8	12.7
*Thyroid bed and distant metastasis*	1	1.6
*Regional lymph node and distant metastases*	6	9.5
*Thyroid bed, regional lymph node and distant metastases*	1	1.6
Histopathologic type		
*Classical type*	45	71.4
*Aggressive type*	18	28.6
BRAF^V600E^ *mutation*		
*Positive*	55	87.3
*Negative*	8	12.7
Characteristic of SUVmax		
*Mean (± SD)*	9.1 (7.1)	
*Median (range)*	6.5 (1.98–36.2)	
*SUVmax < 5*	21	33.3
*5 < SUVmax < 10*	23	36.5
*10 < SUVmax < 20*	13	20.6
*SUVmax > 20*	6	9.5

**Table 2:** The relationship between BRAF^V600E^ mutation and histopathologic types of DTC

Histopathologictype		Classic type		Aggressive type		
		n	%	n	%	P
BRAF^V600E^	Positive	40	88.9%	15	83.3%	
BRAF^V600E^	Negative	5	11.1%	3	16.7%	0.622^a^
Total		45	100%	18	100%	

^a^: Fisher’s exact test

FDG PET detected sites of hypermetabolic recurrence or metastatic disease in most patients 58/63 (92.1%), while the remainder were diagnosed based on contrast-enhanced CT component of the study. Fifty-four/63 (85.7%) patients had regional recurrent disease (thyroid bed or cervical lymph nodes) while 9/63 (14.2%) patients had distant metastatic. Of the latter group, almost all except one had also evidence of local recurrence too. The location of metastatic lesions was mostly in cervical lymph nodes (cervical lymph node alone was seen in 44/63 [69.8%]) while the thyroid bed and distant metastases alone were noted in 2/63 (3.2%) and 1/63 (1.6%), respectively (Table 1).

### Relationship between SUVmax, histopathologic types and BRAF^V600E^ mutation

**Fig. 1:**
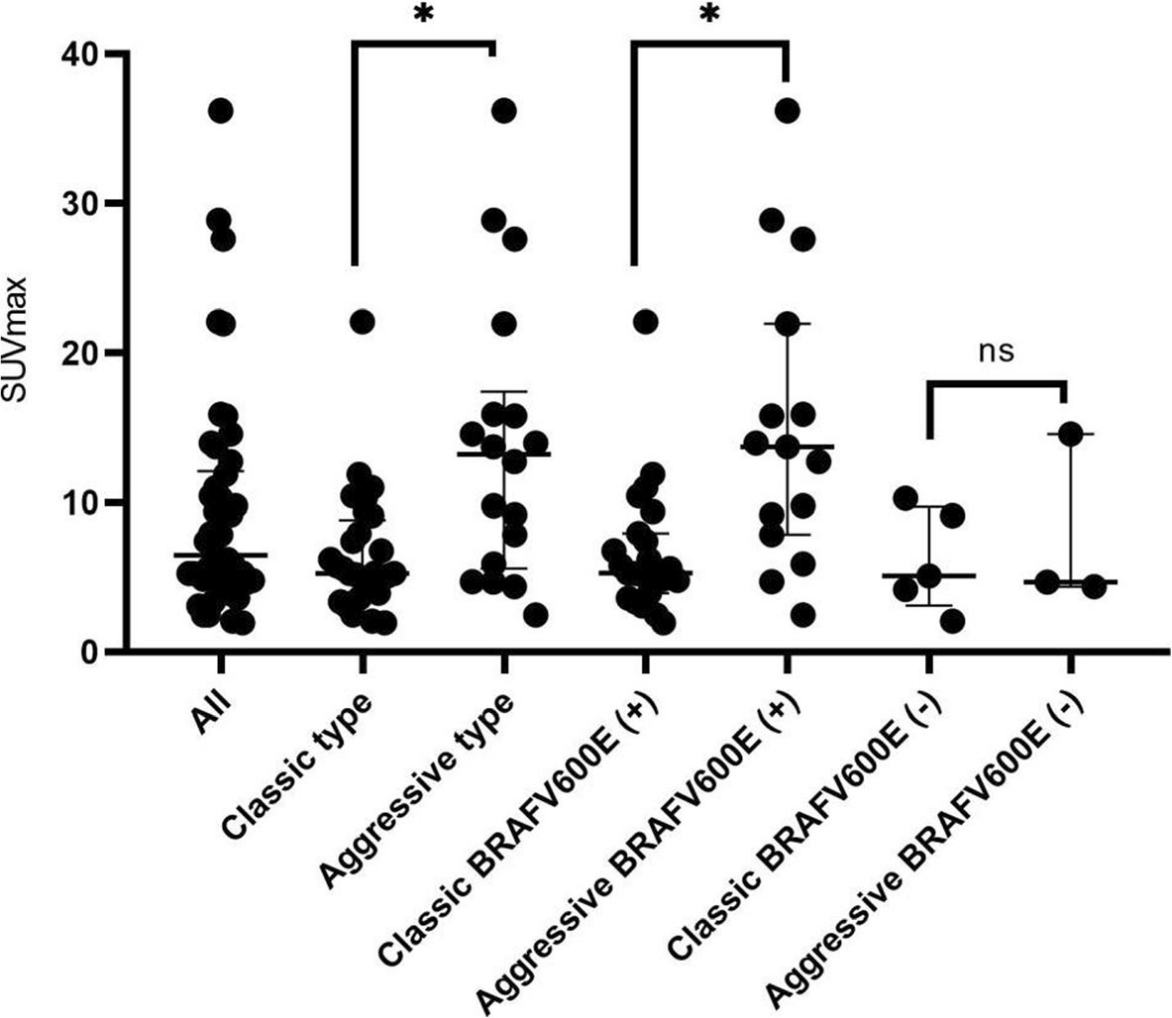
Distribution of SUVmax stratified by histopathologic types of DTC and BRAF^V600E^ mutation status. * significant difference. ns: not significant

The median SUVmax of the BRAF^V600E^ mutation tumors was not significantly higher than those of wild type tumors, 9.5 vs. 6.8, *P* = 0.1 (Fig. 1). In contrast, the median SUVmax of the aggressive histopathologic subtype was significantly higher than the classical subtype, 13.9 vs. 7.2, *P* = 0.0004 (Fig. 1). No significant difference in FDG uptake was found between subgroups of classical DTC with or without BRAF^V600E^ mutation. However, we observed a significant difference of SUVmax in aggressive subtypes with BRAF^V600E^ mutation compared to those with classic subtypes with BRAF^V600E^ mutation, 15.1 vs. 6.2 vs. *P* = 0.0028 (Fig. 1).

**Fig. 2:**
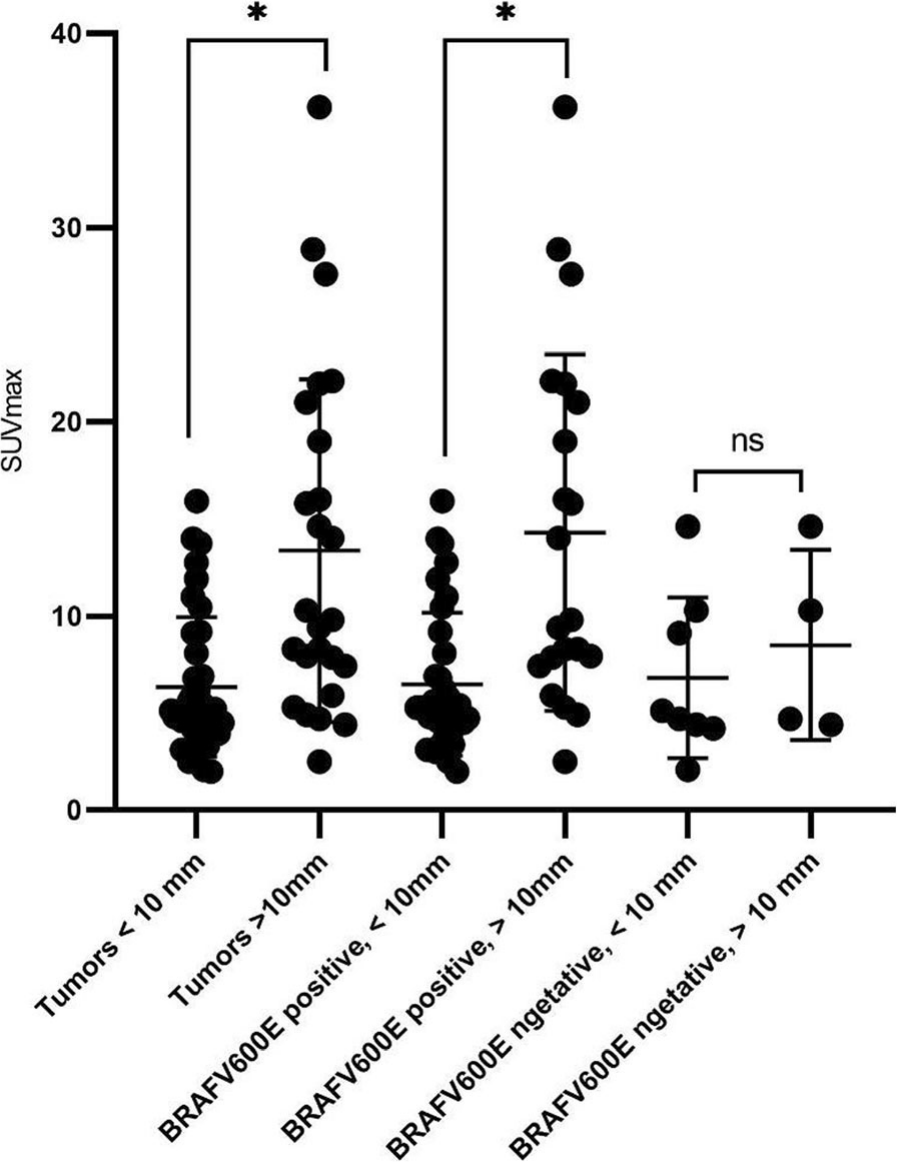
Distribution of SUVmax of DTC tumors stratified by tumor size and BRAF^V600E^ mutation status. * significant difference. ns: not significant

To assess the partial volume effect and SUVmax, we compared the SUVmax between tumors > 10 mm, < 10 mm. SUVmax of lesions > 10 mm was significantly higher than those of lesions < 10 mm, 13.4 vs. 6.3, *P* = 0.0003 (Fig. 2). Consistent results were seen in the subgroup of BRAF^V600E^ positive*,* between tumors larger than 10 mm and smaller than 10 mm, 14.3 vs. 6.5, *P* = 0.0002 (Fig. 2).

**Fig. 3:**
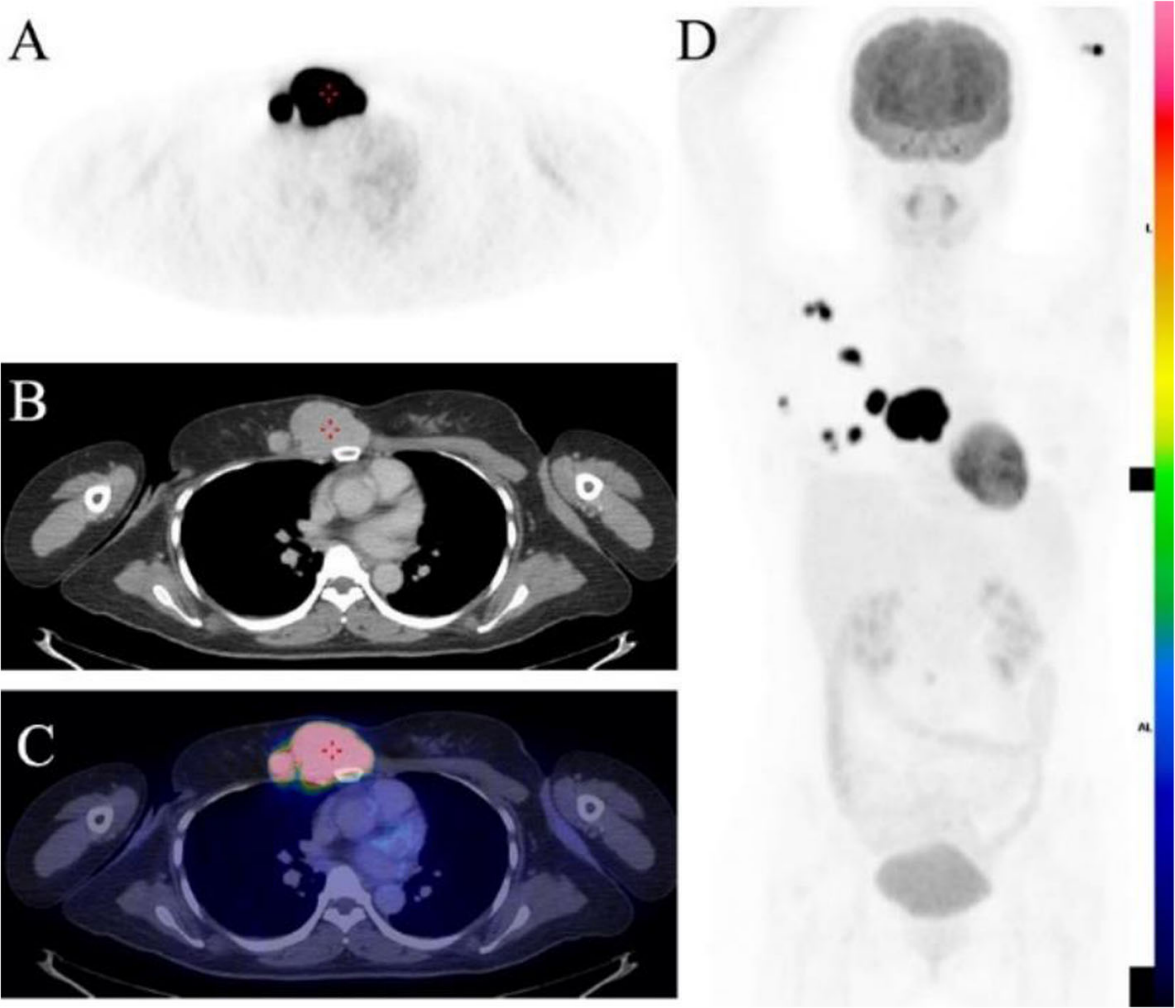
Forty-year-old female with papillary thyroid cancer, aggressive histopathologic type, underwent total thyroidectomy and then received ^131^I therapy with a total dose of 550 mCi. Diagnostic ^131^I whole-body scan after the 3rd treatment showed no abnormal uptake and the patient’s stimulated Tg was > 1000 ng/ml. PET (panel A and D), CT (panel B) and fused PET/CT (panel D) images showed markedly FDG-avid metastatic disease (SUVmax up to 35.55, cross-hair). Surgical resection of that lesion confirmed metastases of papillary thyroid carcinoma, immunohistochemistry and real-time PCR showed BRAF ^V600E^ mutation

**Fig. 4:**
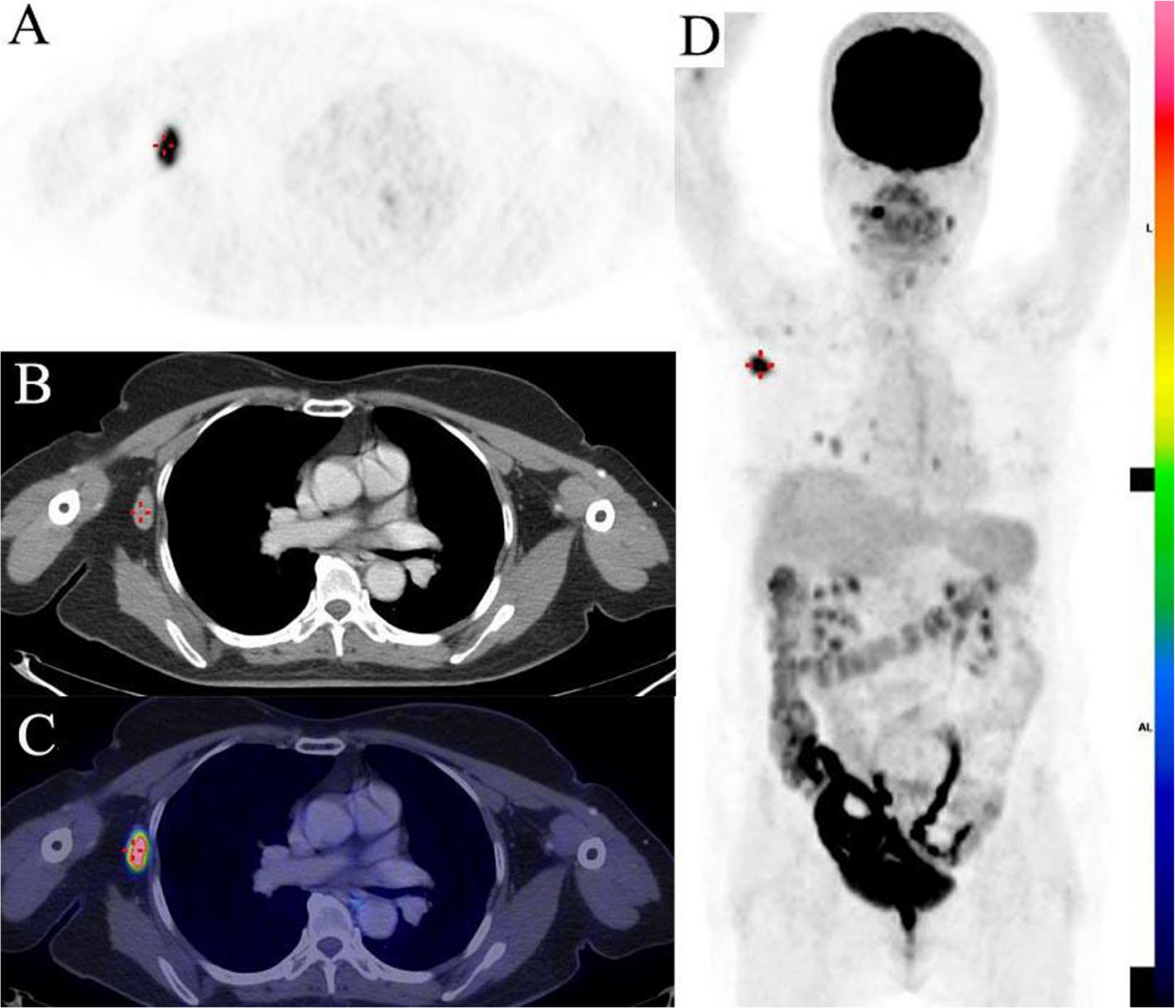
FDG PET/CT with contrast enhancement of a sixty-eight-year-old female with a diagnosis of papillary thyroid carcinoma, previously treated with total thyroidectomy. The patient presented with serum thyroglobulin level of 500 ng/ml and negative post-therapy ^131^I whole-body scan after 3rd treatment. The PET image (panel A and D) illustrated multifocal FDG uptake with the hottest lesion seen in the right axillary region (SUVmax: 13.59) correlated with 11 × 20 mm lymph node on contrast-enhanced CT (panel B, crosshair) and fused PET/CT image (panel C, crosshair). The histopathologic assessment of the lymph node confirmed thyroid carcinoma and immunohistochemistry and real-time PCR was negative for BRAF ^V600E^ mutation

The relationship between clinicopathologic factors and SUVmax is detailed in Table 3. In univariate analysis, FDG uptake was predicted by lesion size, histopathologic type, and distant metastases, *p* = 0.04, *p* = 0.001 and *p* = 0.004 respectively. There was no relationship between SUVmax and BRAF^V600E^ mutation or Tg level on univariate analysis (Figs. 3 and 4).

**Table 3:** Logistic regression analysis for the assessment of association between various factors and SUVmax

Factors	n	Relative risk (95% of CI)	***P***-value
**Histopathologic type**			
Classic type	45	1.153 (1.048–1.269)	0.04*
Aggressive type	18		
**Mutation**			
BRAF^V600E^ (+)	55	1.080 (0.924–1.263)	0.331
BRAF^V600E^ (−)	8		
**Tg (ng/ml)**			
< 250	30	1.066 (0.983–1.157)	0.122
> 250	33		
**Lesion size**			
5–10 mm	38	1.227 (1.084–1.389)	0.001*
> 10 mm	25		
**Recurrence/ metastases**			
Local recurrence	14	0.95 (0.836–1.022)	0.125
Without local recurrence	46		
Lymph node metastases	59	0.923 (0.835–1.020)	0.116
Without lymph node metastases	4		
Distant metastases	9	0.872 (0.793–0.957)	0.004*
Without distant metastases	54		

^*^ significant difference

## Discussions

In this study, we have shown the independent association between FDG avidity and aggressive histopathologic subtypes in patients with recurrent or metastatic RAI-negative disease. The strength of this study was that all patients had histopathologic confirmation of recurrence or metastases and underwent BRAF mutation testing by immunohistochemically and real-time PCR method. In the majority (92%) of the patients, FDG PET detected sites of recurrent disease in RAI-negative patients. This was consistent with the study performed by Rivera et al. which showed that 77% of FDG-avid recurrent or metastases in patients with RAI-refractory disease are of aggressive subtype [19]. However, that study did not describe a comprehensive definition of RAI-refractory state and also did not assess the relationship between SUVmax and histopathologic factors. The underlying mechanism of FDG avidity in aggressive DTC cells is likely the upregulation of glucose transporter-1 (GLUT1) and reduced expression of sodium-iodide symporter (NIS) and this phenomenon proposed that FDG PET/CT is an effective diagnostic tool in aggressive subtypes of DTC [20, 21].

To our surprise, this study did not show the association between BRAF^V600E^ mutation and FDG-avidity. Previous studies have suggested that the SUVmax in BRAF^V600E^ mutation bearing tumors was significantly higher than wild type variants in papillary thyroid cancer (PTC) [22, 23]. It has also been shown that BRAF ^V600E^ mutation is significantly associated with the expression of GLUT-1 and glycolysis in thyroid cancer [24, 25]. The prevalence of BRAF^V600E^ mutation was 87.5% in this study which is consistent with other studies in poorly differentiated thyroid carcinoma and tall cell PTC [26–28]. However, the lower number of patients in the non-BRAF mutation may have limited the statistical power of our study. Furthermore, the result of The Cancer Genome Atlas project indicated that BRAF^V600E^ PTC represents a diverse group of tumors, consisting of multiple molecular subtypes, with variable degrees of thyroid differentiation [29]. Their results also suggested more refined reclassification of thyroid cancers into molecular subtypes that better reflect their underlying signaling and differentiation properties, and potentially better informing the management of these patients. Whether FDG-avidity can be used as an additional clinical tool to further refine the subtypes of tumors with BRAF^V600E^ mutation may warrant further studies. 

Expectedly, our study showed that lesion size is an independent factor of FDG positivity. FDG avidity is strongly dependent on tumor size due to the partial volume effect [30]. Yoon et al. reported in the study of DTC patients that SUVmax of the larger tumors (> 20 mm) was significantly higher than tumors with a smaller size (< 10 mm) [31]. We assumed that the partial volume effect may impact the SUVmax values of the smaller tumor (< 10 mm). When the analysis was performed only in tumors with larger size (> 10 mm), again no significant difference in SUVmax between BRAF^V600E^ mutation and wild type patients was found.

The limitation of our study is the small number of patients in the group with negative BRAF^V600E^ mutation in comparison with the majority with positive mutation which may have limited the statistical power of our study to depict the significant difference in FDG uptake between these two groups. Besides, the limited number of patients in the subgroup with metastatic disease did not allow us to investigate the relationship between BRAF^V600E^ mutation and histopathologic types in these subcategories. As a result, SUVmax values could not be compared to enhance our understanding of the behavior of distant metastatic sites. All patients in our study were completely RAI- negative with varying levels of FDG avidity. However, in clinical practice, a mixed picture could also be seen where both RAI-avid and FDG-positive diseases coexist. Whether these patients still may benefit from RAI treatment or immediately need to be considered for other therapeutic approaches is unclear.

## Conclusions

In patients with RAI-negative and suspected recurrence, FDG PET/CT detects the sites of metastatic disease in the majority of patients. This study suggests that aggressive histopathologic subtypes but not BRAF^V600E^ mutation status are significantly associated with FDG-avidity of the recurrent or metastatic lesions in RAI-negative DTC.

## Data Availability

The data that support the findings of this study are available from the corresponding author upon reasonable request. Pertinent findings: In a retrospective study of 63 patients with recurrent or metastatic RAI-negative DTC, aggressive histopathologic subtypes were independently associated with FDG-avidity of the disease. Implications for patient care: In RAI-negative DTC with suspected recurrence, FDG PET/CT detects sites of recurrent or metastatic disease in most patients. FDG-avidity of the lesions is associated with aggressive histopathologic types hence may require the adoption of a more intense follow-up and treatment strategy.

## Abbreviations

FDG [^18^F]: F-fluoro-2-deoxyglucose
DTC: Differentiated thyroid cancer
PET/CT: Positron emission tomography/computed tomography
RAI: Radioiodine
Tg: Thyroglobulin
EANM: European Association of Nuclear Medicine
NMPs: Nuclear medicine physicians
SUVmax: Maximum standardized uptake value
ROIs: Regions of interest
SPSS: Statistical Package for the Social Sciences
GLUT1: Glucose transporter-1
NIS: Sodium-iodide symporter
PTC: Papillary thyroid cancer
